# Amyloid Beta Leads to Decreased Acetylcholine Levels and Non-Small Cell Lung Cancer Cell Survival via a Mechanism That Involves p38 Mitogen-Activated Protein Kinase and Protein Kinase C in a p53-Dependent and -Independent Manner

**DOI:** 10.3390/ijms25095033

**Published:** 2024-05-05

**Authors:** Hind Al Khashali, Ravel Ray, Ban Darweesh, Caroline Wozniak, Ben Haddad, Stuti Goel, Issah Seidu, Jeneen Khalil, Brooke Lopo, Nayrooz Murshed, Jeffrey Guthrie, Deborah Heyl, Hedeel Guy Evans

**Affiliations:** Chemistry Department, Eastern Michigan University, Ypsilanti, MI 48197, USA; halkhash@emich.edu (H.A.K.); rray9@emich.edu (R.R.); bdarwees@emich.edu (B.D.); cwoznia5@emich.edu (C.W.); bhaddad1@emich.edu (B.H.); sgoel1@emich.edu (S.G.); iseidu@emich.edu (I.S.); jkhalil2@emich.edu (J.K.); blopo@emich.edu (B.L.); nmurshed@emich.edu (N.M.); jguthri7@emich.edu (J.G.); dheylcle@emich.edu (D.H.)

**Keywords:** amyloid beta, acetylcholine, acetylcholinesterase, lung cancer, p38 MAPK, PKC, p53, signaling, cell survival, extracellular

## Abstract

Several studies have shown an inverse correlation between the likelihood of developing a neurodegenerative disorder and cancer. We previously reported that the levels of amyloid beta (Aβ), at the center of Alzheimer’s disease pathophysiology, are regulated by acetylcholinesterase (AChE) in non-small cell lung cancer (NSCLC). Here, we examined the effect of Aβ or its fragments on the levels of ACh in A549 (p53 wild-type) and H1299 (p53-null) NSCLC cell media. ACh levels were reduced by cell treatment with Aβ 1–42, Aβ 1–40, Aβ 1–28, and Aβ 25–35. AChE and p53 activities increased upon A549 cell treatment with Aβ, while knockdown of p53 in A549 cells increased ACh levels, decreased AChE activity, and diminished the Aβ effects. Aβ increased the ratio of phospho/total p38 MAPK and decreased the activity of PKC. Inhibiting p38 MAPK reduced the activity of p53 in A549 cells and increased ACh levels in the media of both cell lines, while opposite effects were found upon inhibiting PKC. ACh decreased the activity of p53 in A549 cells, decreased p38 MAPK activity, increased PKC activity, and diminished the effect of Aβ on those activities. Moreover, the negative effect of Aβ on cell viability was diminished by cell co-treatment with ACh.

## 1. Introduction

Non-small cell lung carcinoma (NSCLC) includes squamous cell carcinoma, large cell carcinoma, and adenocarcinoma, and accounts for ~85% of all lung cancer cases [1,2]. Despite extensive progress in developing treatment strategies that can target NSCLC tumor progression, mortality remains high [3,4].

Cancer and Alzheimer’s disease (AD) have been recently found to share several key pathophysiological features [5,6,7]. Recent reports have shown that patients with AD, considered to be the most common type of dementia, might have a reduced cancer risk and some protection against tumor development [5,6,7,8]. Conversely, relative to patients without a history of cancer, patients who had a cancer diagnosis exhibited a slower cognitive decline and were less likely to develop AD [5,6,7,9,10,11,12,13].

It is known that amyloid-beta (Aβ) (1−40/42) peptides, generated by the cleavage of the amyloid precursor protein (APP) via the amyloidogenic pathway, are linked to the pathology of greater than 20 devastating human diseases including AD and other neurodegenerative disorders [14,15,16,17]. Aβ is approximately 4 kDa and generated from the sequential enzymatic processing of APP by β- and γ-secretase, two membrane-bound endoproteases [18,19]. Different C-terminal heterogeneities of Aβ species exist due to processing by γ-secretase where Aβ 1–40 and Aβ 1–42 represent ~90% and 10% of the isoforms, respectively [18,19,20,21,22,23]. Aβ 1–42 is hydrophobic and fibrillogenic with a high tendency to aggregate into oligomers and is the main form deposited in the brains of people with AD [21,24,25,26]. Aβ 1–40 has a lower tendency to form oligomers [27,28,29]. The amino terminal region of Aβ is relatively hydrophilic, while the carboxyl terminal region is highly hydrophobic, which has been proposed to account for its propensity to aggregate at neutral pH [30,31,32].

Recent reports have shown different levels of secreted Aβ 1–40 and Aβ 1–42 in cancer cells relative to normal controls [5,9,33]. Using human NSCLC cell lines, we have previously reported higher intact Aβ 1–40 and Aβ 1–42 levels in the media of A549 cells (p53 wild-type) than in the media of H1299 cells (p53-null), in part due to the greater proteolytic degradation of Aβ 1–40 and Aβ 1–42 in the media of H1299 cells by matrix metallopeptidase 2 (MMP2) [34].

Neurotransmitters have been reported to play a role in upregulating different aspects of cancer growth, proliferation, progression, and metastasis [35,36]. While the neurotransmitter acetylcholine (ACh) is known to be an important mediator of the parasympathetic nervous system, ACh and cholinergic proteins have been shown to play a vital role in non-neuronal tissues, e.g., lung, promoting cell proliferation and increasing susceptibility to the onset of lung carcinoma [37,38,39,40,41]. The neuronal and non-neuronal cholinergic systems are thought to be differently regulated serving as specific therapeutic targets [37,39,40,42,43]. ACh was shown to be an autocrine growth factor protecting tumors and linked with human cancer risk [4,39,40,42,43,44,45]. Several reports have shown that ACh released from lung cancer cells binds to ionotropic nicotinic (nAChRs) and metabotropic muscarinic (mAChRs) receptors to promote their proliferation [40,41,43,44,45,46,47,48]. The nAChRs are ligand-gated cationic channels and structured as hetero- or homopentamers, while the mAChRs belong to the G-protein coupled receptors family [39,40,41,42,47,49].

Synthesis of ACh occurs in the cytoplasm in a reaction catalyzed by choline acetyltransferase (ChAT) from choline and acetyl-coenzyme A [40,43,44]. ACh is then transported in vesicles to the plasma membrane and released by exocytosis to the extracellular milieu [37,38,39,40,41,50]. ACh is degraded by acetylcholinesterase (AChE) to generate acetate and choline [38,40,50,51,52].

Previously, we reported that insulin-like growth factor binding protein-3 (IGFBP-3) inhibits hyaluronan-CD44 signaling, resulting in increased AChE levels in A549 cell media and apoptosis in a p53-dependent manner [53]. Numerous reports have established the p53 tumor suppressor protein as an important regulator of the expression of a variety of target genes involved in the induction of apoptosis and senescence, cell cycle arrest, and the inhibition of cell proliferation, thereby providing a major barrier to tumorigenesis and controlling a wide range of cellular processes [54,55].

Since H1299 cells are known to be p53-null with minimal expression of AChE compared to A549 (p53 wild-type) cells [4,53,56], we compared the two cell lines to show that treating A549 and H1299 cells with Aβ 1–40, Aβ 1–42, or fragments reduced the levels of ACh in the media of both cell lines in p53-dependent and -independent manner. 

## 2. Results and Discussion

### 2.1. Cell Treatment with Aβ 1–40/42 Led to Decreased ACh Levels in the Media, an Effect Reversed in the Presence of HN

Cancer and AD have been recently found to share several key pathophysiological features and many studies have shown an inverse correlation between the likelihood of developing a neurodegenerative disorder and cancer, with those suffering from a neurodegenerative disorder reported to have a lowered incidence for most cancers [5,6,7]. 

The hydrophilic and more soluble region of Aβ is thought to reside in the amino terminal region, while the carboxy terminal region is relatively hydrophobic and accounts for its tendency to aggregate at neutral pH [5,17,20,21,57,58]. Several reports have shown that Aβ exhibits neurotoxic effects but that amino acids 1–16 in the N-terminal region exhibit neurotrophic effects, while the 25–35 fragment of Aβ is considered as a functional domain of the peptide contributing to its aggregation and neuronal toxicity [15,17,22,59,60]. Several different mechanisms resulting in decreased ACh due to Aβ are likely in operation. 

Humanin (HN) is a small mitochondrial-derived peptide [61,62] that is 24 amino acids long when translated in the cytoplasm [63]. Growing evidence suggests that HN functions as a cyto- and neuroprotective peptide [63,64]. HN has been identified as a binding partner of Aβ likely protecting against its deleterious effects [64,65,66]. We have previously synthesized small peptide segments of Aβ and HN to examine their physical interactions [67], and later showed that HN blocks the aggregation of Aβ induced by AChE [68] and that the interaction of Aβ with HN and AChE is modulated by ATP [69]. We also reported that Aβ levels in the media of NSCLC cells is regulated by matrix metalloproteinases [34]. More recently, we found that the levels of the soluble amyloid precursor protein α (sAPPα) protein are modified by AChE and brain-derived neurotrophic factor in NSCLC cell media [4]. Using synthetic Aβ peptides, Aβ 1–42, Aβ 1–28, and Aβ 25–35 (but not Aβ 1–16) were found to reduce the levels of ACh using a mouse cell line derived from basal forebrain cholinergic neurons [70]. Aβ fragments, including Aβ 1–42, Aβ 1–40, Aβ1–28 and Aβ 25–35, were also shown to block ACh release from rat hippocampal slices [71].

**Figure 1:**
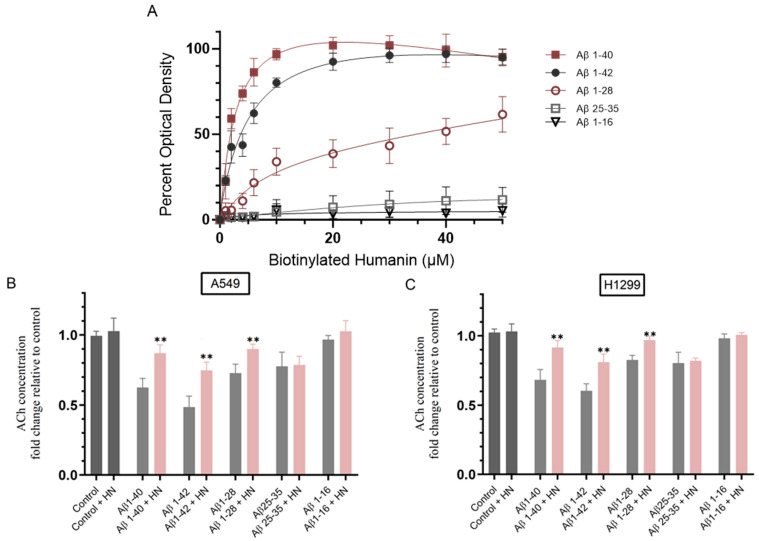
Binding of HN to Aβ reverses the reduction in ACh levels in the media observed upon cell incubation with only Aβ. (**A**) Interaction of Aβ 1–40/42 or fragments with biotinylated-HN. Aβ (10 μM) was bound to the wells. Increasing concentrations of biotinylated-HN were then added to the wells and processed as described in the Materials and Methods Section. Optical density measurements (450 nm) were normalized by expressing each point in relation to the best fitted Emax value (set to 100%). The data were then plotted as a function of increasing biotinylated-HN concentrations. The data were fitted to a single binding site model with a nonlinear regression curve fitting approach and plotted as the mean ± S.D. of three independent trials, each performed in triplicate using the GraphPad Prism 10.1.1 software. (**B**,**C**) Cells (0.2 × 10) were grown in 10% FBS-supplemented media for 24 h. The following day, the cell monolayers were incubated in serum-free media overnight, then treated for 72 h with Aβ 1–40/42 or fragments (10 μM), HN (10 μM), or in combination. The levels of ACh in the media were then measured as described in the Materials and Methods Section. Data from five independent assays, each carried out in triplicate, were averaged, normalized, and expressed as fold change relative to untreated cells (control) using the GraphPad 10.1.1 software. The graphs summarize the results expressed as means ± SD (n = 5). Asterisks indicate a statistically significant difference from the corresponding sample in the absence of HN while absence of asterisks indicates no significance, Mann–Whitney test, ** *p* < 0.01.

To test the effect of incubating A549 and H1299 cells with Aβ 1–40/42 or fragments on the levels of ACh in the media, we first examined the binding of the different Aβ fragments to HN, reported to function as a natural broad spectrum cytoprotective peptide in part due to its ability to directly bind to Aβ, thereby blocking its toxicity [64,69,72,73,74]. We reasoned that the ability of HN to nullify the effect of different Aβ fragments might provide further support for the function of certain Aβ segments in modulating ACh levels. We chose a concentration of 10 μM Aβ since compared to buffer control, significant differences were observed and as this concentration has been previously used by several investigators. Aβ was bound to the wells (Figure 1A), and then, increasing concentrations of biotinylated-HN were added to the wells and processed as described in the Materials and Methods Section. HN was able to bind both Aβ 1–40/42 in accord with our previous reports [67,68,69]. HN bound Aβ 1–40/42 more effectively than Aβ 1–28, while the binding to Aβ 25–35 was significantly abolished, and no binding was found between HN and Aβ 1–16 (Figure 1A). Amino acid residues 17–28 of Aβ were previously reported by proteolytic epitope excision and extraction in addition to affinity-mass spectrometric data analysis to be involved in direct interactions between HN and Aβ40 [75]. Our results show (Figure 1A) that binding of HN to Aβ 1–28 was less effective than that to Aβ 1–40/42 despite the inclusion of residues 17–28 in this segment, perhaps suggesting that the complete Aβ sequence is necessary for optimal HN-Aβ interactions. Aβ 25–35 lacks amino acids 17–24, which likely explains its greatly diminished ability to interact with HN. No binding between HN and Aβ 1–16 was observed which was expected since this fragment lacks residues 17–28 of Aβ.

To examine the effect of Aβ 1–40/42 or fragments on the levels of ACh in the media, A549 and H1299 cells were grown in 10% FBS-supplemented media for 24 h then incubated in serum-free media overnight. The cells were then treated for 72 h with Aβ 1–40/42 or fragments, HN, or in combination, and the levels of ACh in the media were then measured as described in the Materials and Methods Section (Figure 1B,C). Relative to control untreated cells, no effect on the levels of ACh was found upon incubation with HN. Cell treatment with Aβ 1–40 resulted in ~1.60-fold reduction in the levels of ACh in the media of A549 cells (Figure 1B) and ~1.45-fold decrease in the media of H1299 cells (Figure 1C). Co-treatment of Aβ 1–40 with HN decreased the levels of ACh in A549 media by ~1.15-fold and by ~1.05-fold in the media of H1299 cells. Cell treatment with Aβ 1–42 was more effective than treatment with Aβ 1–40 in reducing the levels of ACh in the media and led to ~2.00-fold reduction in the levels of ACh in the media of A549 cells (Figure 1B) and ~1.65-fold decrease in the media of H1299 cells (Figure 1C). Co-treatment of Aβ 1–42 with HN decreased the levels of ACh in A549 media by ~1.35-fold (Figure 1B) and by ~1.25-fold in the media of H1299 cells (Figure 1C). Reductions in the levels of ACh in the media were more modest upon cell incubation with Aβ 1–28 and resulted in ~1.35-fold reduction in A549 cell media (Figure 1B) and ~1.20-fold in the media of H1299 cells (Figure 1C). Co-treatment of HN with Aβ 1–28 decreased the levels of ACh in A549 media by ~1.10-fold and abolished the effects observed by using only Aβ 1–28 in the media of H1299 cells. Cell incubation with Aβ 25–35 resulted in comparable reductions in the levels of ACh in the media to those found with Aβ 1–28 treatment, and no further effects on those levels were observed upon co-treatment of Aβ 25–35 with HN in either cell lines (Figure 1B,C). A549 or H1299 cell incubation with Aβ 1–16 with or without HN had no effect on the levels of ACh in the media (Figure 1B,C). 

Collectively, these results suggest that the levels of ACh in the media of A549 and H1299 cells are not altered by incubation with Aβ 1–16, but that they are more effectively reduced by cell treatment with Aβ 1–42 followed by cell treatment with Aβ 1–40 and then with Aβ 1–28 and Aβ 25–35. In both cell lines, HN was able to reverse the effects of Aβ 1–40/42 and Aβ 1–28 only (Figure 1B,C), which correlated with its more effective binding to those segments (Figure 1A). 

### 2.2. AChE and p53 Activities Increased upon Treatment of A549 Cells with Aβ Fragments, Except Aβ 1–16, While Co-treatment of Aβ 1–40/42/28 with HN Blocked This Effect

**Figure 2:**
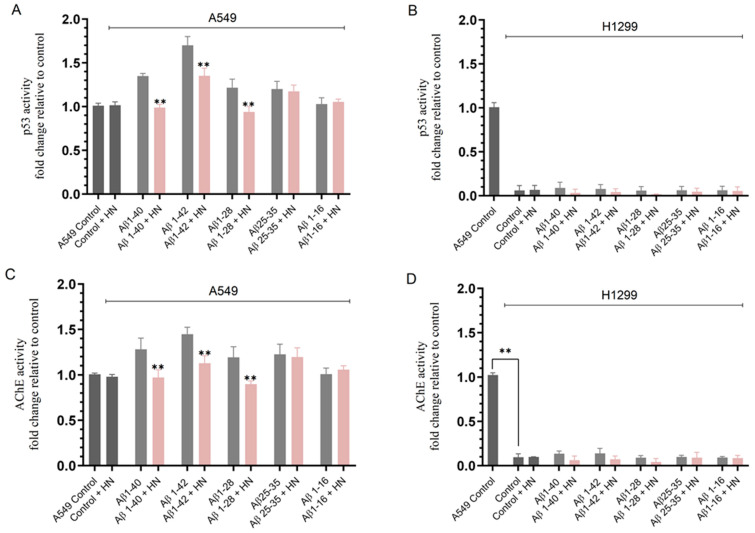
Treatment of A549 cells with all Aβ fragments, except for Aβ 1–16, increased the activity of p53 and AChE, an effect diminished upon binding of HN to Aβ 1–40/42/28. Cells (0.2 × 10) were grown in 10% FBS-supplemented media for 24h. The following day, the cell monolayers were incubated in serum-free media overnight, then treated for 72 h with Aβ 1–40/42 or fragments (10 μM), HN (10 μM), or in combination. The activity of p53 in lysates of A549 (**A**) and H1299 (**B**) cells and the activity of AChE in the media of A549 (**C**) and H1299 (**D**) cells were then measured as described in the Materials and Methods Section. Data from three independent assays, each carried out in triplicate, were averaged, normalized, and expressed as fold change relative to untreated A549 cells (**A**–**D**, A549 control) using the GraphPad 10.1.1 software. The graphs summarize the results expressed as means ± SD (n = 3). Asterisks indicate a statistically significant difference from the corresponding sample in the absence of HN, while absence of asterisks indicates no significance, Mann–Whitney test, ** *p* < 0.0l.

Levels of ACh, known to act as a growth factor for human NSCLC, are thought to be elevated in lung cancer via mechanisms that may include the upregulation of ACh signaling by decreasing the levels and activity of AChE [37,38,39,40,41,48,49,76,77]. Aβ 1–40 was found to block ACh release by activation of AChE in neuronal systems, and both Aβ 1–40 and Aβ 1–28 were shown to increase AChE activity in a panel of human squamous cell carcinoma (SCC-L) cell lines [40,41,47]. Earlier, we reported that IGFBP-3 treatment of A549 cells (p53 wild-type), but not H1299 cells (p53-null), transfected with either p53 siRNA or with AChE siRNA, resulted in decreased AChE levels and activity in the media, decreased apoptosis, and increased cell viability [53]. In this study, we tested the effect of added Aβ 1–40/42 or fragments on the activity of p53 and AChE (Figure 2). Cells were grown in FBS-supplemented media for 24 h, serum-starved overnight, then treated for 72 h with Aβ 1–40/42 or fragments, HN, or in combination (Figure 2). The activity of p53 in the cell lysates and the activity of AChE in the media were then measured as described in the Materials and Methods Section. 

Relative to control untreated A549 cells, no effect on the activity of p53 was observed upon incubation with HN (Figure 2A). A549 cell treatment with Aβ 1–40 and Aβ 1–42 increased the activity of p53 by ~1.35-fold and ~1.70-fold, respectively, while incubation with Aβ 1–28 or Aβ 25–35 led to ~1.22-fold increase in p53 activity, and no effect was found when using Aβ 1–16 (Figure 2A). Relative to samples without HN, A549 cell co-treatment with HN and Aβ 1–40, Aβ 1–42, or Aβ 1–28 decreased the activity of p53 by ~1.35-fold, ~1.25-fold, and ~1.30-fold, respectively (Figure 2A). Addition of HN to A549 cells in the presence of Aβ 25–35 or Aβ 1–16 did not alter the p53 activity compared to cell treatment in the absence of HN (Figure 2A). As expected, there was no detection of p53 activity in H1299 cells since they are known to be p53-null (Figure 2B). 

The trends observed for the AChE activity in A549 cell media were comparable to those found for the activity of p53 under the same conditions (Figure 2C). No effect on the activity of AChE was observed upon the incubation of A549 cells with HN relative to control untreated cells (Figure 2C). A549 cell treatment with Aβ 1–40 or Aβ 1–42 increased the AChE activity by ~1.25-fold and ~1.45-fold, respectively, while incubation with Aβ 1–28 or Aβ 25–35 led to ~1.18-fold increase in AChE activity, and no effect was found when using Aβ 1–16 (Figure 2C). Relative to samples without HN, A549 cell co-treatment with HN and Aβ 1–40, Aβ 1–42, or Aβ 1–28 decreased the activity of AChE by ~1.32-fold, ~1.28-fold, and ~1.32-fold, respectively (Figure 2C). Addition of HN to A549 cells in the presence of Aβ 25–35 or Aβ 1–16 did not alter the AChE activity compared to cell treatment in the absence of HN (Figure 2C). Minimal detection of AChE activity was found in H1299 cell media (Figure 2D), a result consistent with our previous reports [4,53,68,69]. 

The reduced activation of p53 (Figure 2A) and AChE (Figure 2C) by addition of HN to A549 cells treated with Aβ 1–40, Aβ 1–42, or Aβ 1–28 correlated with increased ACh levels in the media (Figure 1B), a finding likely due to a decrease in AChE activity. However, the increased ACh levels in H1299 cell media (Figure 1C) upon incubation of cells with HN and Aβ 1–40, Aβ 1–42, or Aβ 1–28 cannot be accounted for by the possible effect of Aβ on either p53 or AChE since H1299 cells are p53-null with minimal expression of AChE relative to A549 cells. 

It is unclear whether Aβ treatment alters the expression and/or release of HN in NSCLC, and research into this area is currently being carried out in our laboratory.

### 2.3. Knockdown of p53 in A549 Cells Led to Increased ACh Levels, Decreased AChE Activity, and Reduced Aβ Effects on Both ACh and AChE

**Figure 3:**
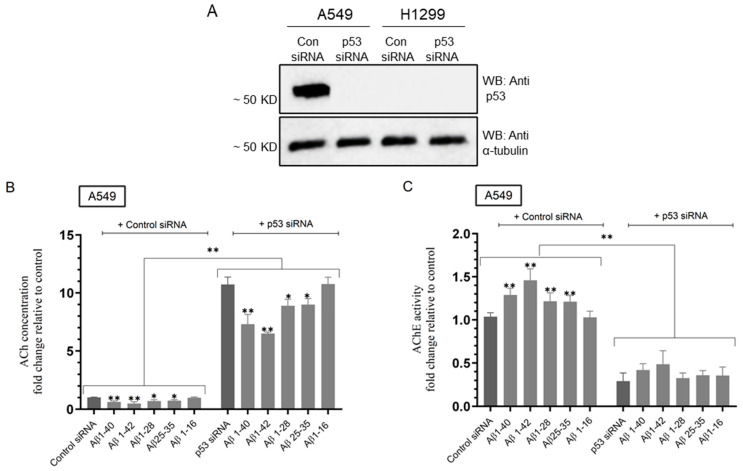
Transfection of A549 cells with p53 siRNA led to enhanced ACh levels and diminished AChE activity in the media, and reduced Aβ effects on both ACh and AChE. Cells (0.2 × 10) were grown in 10% FBS-supplemented media for 24 h and then serum-starved overnight. The cells were then treated as indicated for 72 h with control siRNA or p53 siRNA. The same concentration of total protein (15 μL of 600 μg/mL) of the cell lysates (**A**) was used for Western blotting using the indicated antibodies. As a loading control, anti α-tubulin antibodies were used. Transfected cells were treated for 72 h with Aβ 1–40/42 or fragments (10 μM), and then, the levels of ACh (**B**) and activity of AChE in the media (**C**) of A549 cells were then measured as described in the Materials and Methods Section. Data from five independent assays, each carried out in triplicate, were averaged, normalized, and expressed as fold change relative to control siRNA untreated with Aβ fragments using the GraphPad 10.1.1 software. The graphs summarize the results expressed as means ± SD (n = 5). Asterisks indicate a statistically significant difference from cells treated with control or p53 siRNA. Absence of asterisks indicates no significance, Mann–Whitney test. Statistical differences between different groups were analyzed by an ordinary one-way analysis of variance (ANOVA) followed by Tukey’s post hoc multiple comparison test, * *p* < 0.05, ** *p* < 0.01.

Our results (Figure 2) showed that AChE and p53 activities increased upon treatment of A549 cells with Aβ fragments, except Aβ 1–16. Previously, we reported that treatment of A549 cells, transfected with either p53 siRNA or with AChE siRNA, with IGFBP-3 led to decreased AChE levels and activity in the media [53]. We therefore set out to examine the effect of p53 knockdown in A549 cells on the levels of ACh and activity of AChE upon treatment with Aβ 1–40/42 or fragments (Figure 3).

A549 cells were grown in FBS-supplemented media for 24 h then serum-starved overnight. The cells were then treated for 72 h with control siRNA or p53 siRNA and Aβ 1–40/42 or fragments as indicated (Figure 3), and then, the levels of ACh and activity of AChE in the media were measured as described in the Materials and Methods Section. Knockdown of p53 in A549 cells resulted in ~10.75-fold increase in ACh levels in the media (Figure 3A,B). Relative to A549 cells transfected with p53 siRNA, treatment with Aβ 1–40 or Aβ 1–42, led to ~1.47-fold and ~1.65-fold reduction in ACh levels in the media, respectively, while ~1.20-fold reduction was found when incubating cells with either Aβ 1–28 or Aβ 25–35, and no effects were observed when using Aβ 1–16 (Figure 3B). Compared to untreated transfected cells, the effects of Aβ on the levels of ACh in the media of A549 cells transfected with p53 siRNA were less pronounced than those found in A549 cells transfected with control siRNA [Aβ 1–40 or Aβ 1–42, led to ~1.65-fold and ~2.00-fold reduction, respectively, while ~1.45-fold reduction was found with either Aβ 1–28 or Aβ 25–35, and no change was observed with Aβ 1–16 (Figure 3B)]. The activity of AChE in the media showed an opposite trend (Figure 3C) to those observed for the levels of ACh (Figure 3B). The AChE activity decreased by ~3.40-fold upon transfection of A549 cells with p53 siRNA compared to cells transfected with control siRNA, results consistent with our previous finding [53]. Treatment of A549 cells transfected with control siRNA with Aβ 1–40 or Aβ 1–42 increased the AChE activity by ~1.28-fold and ~1.45-fold, respectively, while incubation with Aβ 1–28 or Aβ 25–35 led to ~1.20-fold increase in AChE activity, and no effect was found when using Aβ 1–16 (Figure 3C). These results are comparable to those measured in Figure 2C. Compared to untreated A549 cells transfected with p53 siRNA, the effects of Aβ on the activity of AChE in the media were not significant (Figure 3C), possibly suggesting that Aβ regulates AChE in a p53-dependent manner. 

### 2.4. The Ratio of Phospho/Total p38 MAPK Increased upon A549 and H1299 Cell Treatment with Aβ While Inhibiting p38 MAPK Activity Decreased p53 Activity in A549 Cells and Increased ACh Levels in the Media of Both Cell Lines

The MAPK subfamilies include the extracellular signal-regulated kinase (ERK), c-Jun-N-terminal kinase (JNK), and p38 mitogen-activated protein kinase (p38 MAPK) [78,79]. The response of p38 MAPK to stress stimuli is well documented, and increased apoptosis has been reported for the p38 kinase pathways [78]. Aβ has been shown to activate p38 MAPK in cultured neurons, and the deletion of p38α MAPK in neurons was found to decrease Aβ load in an Alzheimer mouse model [80]. Activation of p38 occurs by dual phosphorylation at amino acid residues Thr180 and Tyr182 in the activation loop [78]. Evidence suggests that p38 MAPK can function as a tumor suppressor by inducing p53 phosphorylation, causing p53-dependent growth arrest, activating apoptosis, and negatively regulating the cell cycle, while inhibiting p38 MAPK activity prevented p53 phosphorylation and blocked apoptosis [78,79]. Phosphorylation of several N-terminal residues of p53, including Ser15, Ser20, and Ser37, has been reported to be important for stabilizing the protein [81]. Following exposure of MCF-7 cells to UV radiation, p38 kinase was found to phosphorylate p53 at Ser33 and Ser46 and led to activated p53-mediated transcription and apoptosis [81]. Moreover, substitution of Ser33 and Ser46 with alanine inhibited p53-mediated apoptosis [81]. ACh was reported to abolish p38-MAPK phosphorylation and signaling, and treatment with Ach or the p38 MAPK inhibitor SB203580 attenuated TNF-α induced apoptosis, leading to protection of cardiomyocytes [82]. 

**Figure 4:**
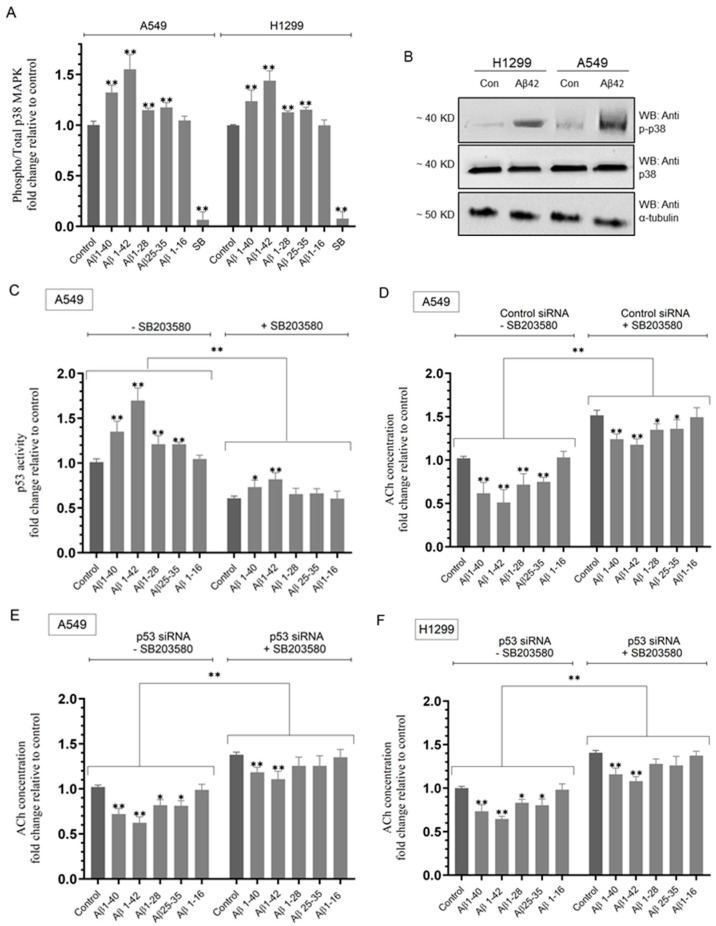
Treatment of cells with Aβ led to increased phospho/total p38 MAPK ratio, while blocking p38 MAPK activity with SB203580 decreased p53 activity in A549 cells and increased ACh levels in the media of both cell lines. Cells (0.2 × 10) were grown in 10% FBS-supplemented media for 24 h and then serum-starved overnight. The cells were then treated as indicated for 72 h with Aβ 1–40/42 or fragments (10 μM) ± SB203580 (SB, 20 μM), and then, the p38 MAPK assay (**A**) and Western blotting (**B**) were carried out (Materials and Methods). The p53 activity was measured in A549 cells (**C**). Cells transfected with either control or p53 siRNA were treated for 72 h with Aβ 1–40/42 or fragments ± SB203580; then, the levels of ACh (**D**–**F**) were measured as described in the Materials and Methods Section. Data from five independent assays, each carried out in triplicate, were averaged, normalized, and expressed as fold change relative to the control of each cell line (**A**) or to control in the absence of SB203580 (**C**–**F**) using the GraphPad 10.1.1 software. The graphs summarize the results expressed as means ± SD (n = 5). Asterisks indicate a statistically significant difference from control. Absence of asterisks indicates no significance, Mann–Whitney test. Statistical differences between different groups were analyzed by an ordinary one-way analysis of variance (ANOVA) followed by Tukey’s post hoc multiple comparison test, * *p* < 0.05, ** *p* < 0.01.

To examine whether the effects of Aβ on the levels of ACh in the media are dependent on p53 or whether they can also be p53-independent, we tested the hypothesis that Aβ activates p38 MAPK, leading to decreased ACh levels in both a p53-dependent and p53-independent manner. Cells were grown in FBS-supplemented media for 24 h and then serum-starved overnight. The cells were then transfected with either control or p53 siRNA and treated as indicated for 72 h with Aβ 1–40/42 or fragments in the absence or presence of the p38 MAPK inhibitor, SB203580, and then, the p38 MAPK activity, p53 activity, and ACh levels (Figure 4) were measured (Materials and Methods). 

A549 cell treatment with Aβ 1–40 led to ~1.32-fold increase in the ratio of phospho/total p38 MAPK while that increase was ~1.55-fold upon treatment with Aβ 1–42 (Figure 4A,B). A549 cell treatment with either Aβ 1–28 or Aβ 25–35 led to a more modest increase (~1.15-fold) in this ratio, while no effects were observed when cells were treated with Aβ 1–16. Similar trends were observed when using H1299 cells (Figure 4A,B). H1299 cell treatment with Aβ 1–40 led to ~1.23-fold increase in the ratio of phospho/total p38 MAPK and ~1.45-fold increase upon treatment with Aβ 1–42 (Figure 4A,B). Similar to the results obtained with A549, H1299 cell treatment with either Aβ 1–28 or Aβ 25–35 led to a more modest increase (~1.15-fold) in the phospho/total p38 MAPK ratio, and no effects were observed when H1299 cells were treated with Aβ 1–16 (Figure 4A). These results show that Aβ leads to an increased ratio of phospho/total p38 MAPK in both A549 (p53-positive) and H1299 (p53-null) cell lines (Figure 4A,B) and that this activation is independent of p53. 

We next investigated the effects of Aβ peptides on the activity of p53 in A549 cells in the absence or presence of the p38 MAPK inhibitor, SB203580 (Figure 4C). Compared to untreated A549 cells, treatment with SB203580 resulted in ~1.65-fold decrease in the activity of p53 (Figure 4C). Relative to control A549 cells in the absence of SB203580, the p53 activity increased by ~1.35-fold with Aβ 1–40, ~1.70-fold with Aβ 1–42, ~1.20-fold with either Aβ 1–28 or Aβ 25–35, and no difference was found with Aβ 1–16 treatment (Figure 4C). These effects were less pronounced upon A549 cell treatment with SB203580 (Figure 4C). Relative to control A549 cells in the presence of SB203580, the p53 activity increased by ~1.20-fold with Aβ 1–40 and ~1.35-fold with Aβ 1–42, while no significant difference was observed with either Aβ 1–28, Aβ 25–35, or Aβ 1–16 treatment (Figure 4C). This finding suggests that inhibiting p38 MAPK leads to decreased p53 activation and that the ability of Aβ to activate p53 is diminished, but not abolished, upon blocking p38 MAPK with SB203580 in A549 cells (Figure 4C).

We next examined the effects of p53 knockdown on the levels of ACh in the media upon cell treatment with Aβ 1–40/42 or fragments in the absence or presence of the p38 MAPK inhibitor, SB203580 (Figure 4D–F). Compared to untreated A549 cells transfected with control siRNA, treatment with SB203580 resulted in ~1.50-fold increase in the levels of ACh (Figure 4D). Relative to A549 cells transfected with control siRNA in the absence of SB203580, the levels of ACh decreased by ~1.65-fold with Aβ 1–40, ~2.00-fold with Aβ 1–42, ~1.35-fold with either Aβ 1–28 or Aβ 25–35, and no difference was found with Aβ 1–16 treatment (Figure 4D). These effects were less pronounced upon treatment of A549 cells transfected with control siRNA with SB203580 (Figure 4D). Relative to control A549 cells in the presence of SB203580, the concentration of ACh in the media decreased by ~1.24-fold with Aβ 1–40, ~1.30-fold with Aβ 1–42, and ~1.12-fold with either Aβ 1–28 or Aβ 25–35, with no observed effect upon cell treatment with Aβ 1–16 (Figure 4D). These results suggest that inhibiting p38 MAPK leads to increased ACh levels and that the ability of Aβ to decrease those levels is diminished, but not abolished, upon blocking p38 MAPK with SB203580 in A549 cells (Figure 4D), highlighting a possible role for the kinase in the Aβ-dependent decrease of ACh in the media of A549 cells. 

More modest effects but similar trends were found in A549 cells transfected with p53 siRNA (Figure 4E). Compared to untreated A549 cells transfected with p53 siRNA, treatment with SB203580 resulted in ~1.35-fold increase in the levels of ACh (Figure 4E). Relative to A549 cells transfected with p53 siRNA in the absence of SB203580, the levels of ACh decreased by ~1.35-fold with Aβ 1–40, ~1.60-fold with Aβ 1–42, ~1.20-fold with either Aβ 1–28 or Aβ 25–35, and no difference was found with Aβ 1–16 treatment (Figure 4E). These effects were less pronounced upon transfection of A549 cells with p53 siRNA and treatment with SB203580 (Figure 4E). Relative to control A549 cells in the presence of SB203580, the concentration of ACh in the media decreased by ~1.15-fold with Aβ 1–40, ~1.20-fold with Aβ 1–42, and no significant difference was observed with either Aβ 1–28, Aβ 25–35, or Aβ 1–16 treatment (Figure 4E). These results were comparable to those obtained using H1299 cells transfected with p53 siRNA (Figure 4F). 

Collectively, these results clearly show that p38 MAPK and p53 are important regulators of the levels of ACh. However, the observation that the levels of ACh are still reduced upon treatment with Aβ 1–40 or Aβ 1–42 in the presence of the p38 MAPK inhibitor, SB203580, in both cell lines and by knockdown of p53 in A549 cells suggests that other pathways are involved in the regulation of the concentration of ACh by Aβ 1–40/42 in addition to p53 and p38 MAPK. 

### 2.5. The Activity of PKC Decreased upon A549 and H1299 Cell Treatment with Aβ While Inhibiting PKC with Chelerythrine Increased p53 Activity in A549 Cells and Decreased ACh Levels in the Media of Both Cell Lines

Reports have previously shown that protein kinase C (PKC) can phosphorylate the C-terminal regulatory domain of p53 leading to its ubiquitination and degradation [83,84,85]. Treatment of Dalton lymphoma cells with chelerythrine or staurosporine led to increased p53-dependent apoptotic pathways [86].

**Figure 5:**
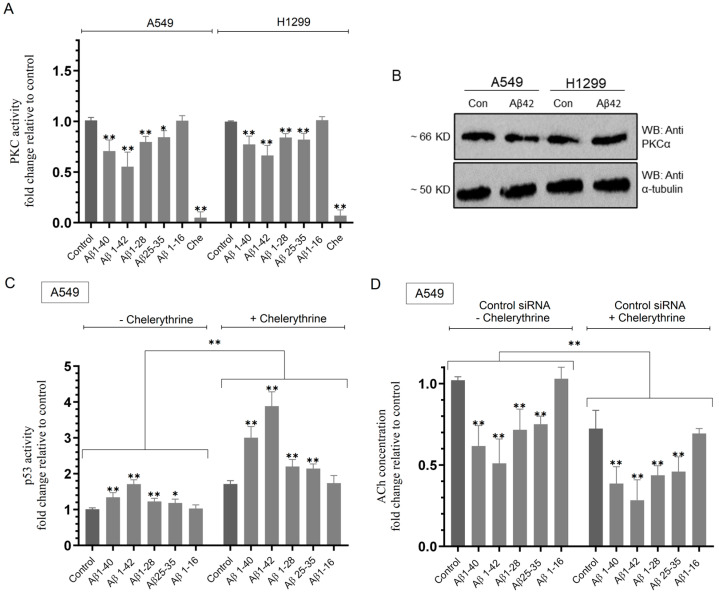

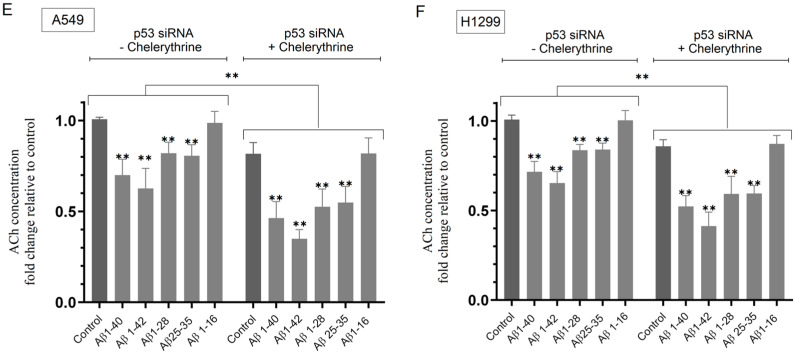
Treatment of cells with Aβ led to decreased PKC activity, while blocking PKC activity with chelerythrine increased p53 activity in A549 cells and decreased ACh levels in the media of both cell lines. Cells (0.2 × 10^5^) were grown in 10% FBS-supplemented media for 24 h and then serum-starved overnight. The cells were then treated as indicated for 72h with Aβ 1–40/42 or fragments (10 μM) ± the PKC inhibitor (chelerythrine, 7.5 μM), and then, the PKC activity assay (**A**) and Western blotting (**B**) were carried out (Materials and Methods). The p53 activity was measured in A549 cells (**C**). Cells transfected with either control or p53 siRNA were treated for 72 h with Aβ 1–40/42 or fragments ± chelerythrine, and then, the levels of ACh (**D**–**F**) were measured as described in the Materials and Methods Section. Data from five independent assays, each carried out in triplicate, were averaged, normalized, and expressed as fold change relative to the control of each cell line (**A**) or to control in the absence of chelerythrine (**C**–**F**) using the GraphPad 10.1.1 software. The graphs summarize the results expressed as means ± SD (n = 5). Asterisks indicate a statistically significant difference from control. Absence of asterisks indicates no significance, Mann–Whitney test. Statistical differences between different groups were analyzed by an ordinary one-way analysis of variance (ANOVA) followed by Tukey’s post hoc multiple comparison test, * *p* < 0.05, ** *p* < 0.01.

To test the hypothesis that Aβ inhibits PKC leading to decreased ACh levels in both a p53-dependent and p53-independent manner, cells were grown in 10% FBS-supplemented media for 24 h and then serum-starved overnight. Cells (untransfected or transfected with control or p53 siRNA) were then treated as indicated for 72 h (Figure 5) with Aβ 1–40/42 or fragments in the absence or presence of the PKC inhibitor, chelerythrine, and then, the activities of PKC and p53 and ACh levels in the media were measured as described in the Materials and Methods Section. 

A549 cell treatment with Aβ 1–40 led to ~1.40-fold decrease in the activity of PKC while that decrease was ~1.80-fold upon treatment with Aβ 1–42 (Figure 5A,B). A549 cell treatment with either Aβ 1–28 or Aβ 25–35 led to a more modest decrease (~1.20-fold) in this activity, while no effects were observed when cells were treated with Aβ 1–16. Similar trends were observed when using H1299 cells (Figure 5A). H1299 cell treatment with Aβ 1–40 led to ~1.30-fold decrease in the PKC activity and ~1.50-fold decrease upon treatment with Aβ 1–42 (Figure 5A,B). Similar to the results obtained with A549, H1299 cell treatment with either Aβ 1–28 or Aβ 25–35 led to a more modest decrease (~1.20-fold) in the activity of PKC, and no effects were observed when H1299 cells were treated with Aβ 1–16 (Figure 5A). These results show that Aβ leads to the decreased activation of PKC in both A549 (p53-positive) and H1299 (p53-null) cell lines (Figure 5A,B) and that this effect is independent of p53. 

We next investigated the effects of Aβ peptides on the activity of p53 in A549 cells in the absence or presence of the PKC inhibitor, chelerythrine (Figure 5C). Compared to untreated A549 cells, treatment with chelerythrine resulted in ~1.70-fold increase in the activity of p53 (Figure 5C). Relative to control A549 cells in the absence of chelerythrine, the p53 activity increased by ~1.35-fold with Aβ 1–40, ~1.70-fold with Aβ 1–42, ~1.20-fold with either Aβ 1–28 or Aβ 25–35, and no difference was found with Aβ 1–16 treatment (Figure 4C). These effects were more pronounced upon A549 cell treatment with chelerythrine (Figure 5C). Relative to control A549 cells in the presence of chelerythrine, the p53 activity increased by ~1.75-fold with Aβ 1–40, ~2.25-fold with Aβ 1–42, ~1.30-fold with either Aβ 1–28 or Aβ 25–35, and no difference was found with Aβ 1–16 treatment (Figure 5C). This finding suggests that inhibiting PKC leads to increased p53 activation and that the ability of Aβ to activate p53 is enhanced upon blocking the activity of PKC with chelerythrine in A549 cells (Figure 5C).

We next examined the effects of p53 knockdown on the levels of ACh in the media upon cell treatment with Aβ 1–40/42 or fragments in the absence or presence of the PKC inhibitor, chelerythrine (Figure 5D–F). Compared to untreated A549 cells transfected with control siRNA, treatment with chelerythrine resulted in ~1.40-fold decrease in the levels of ACh (Figure 5D). Relative to A549 cells transfected with control siRNA in the absence of chelerythrine, the levels of ACh decreased by ~1.60-fold with Aβ 1–40, ~2.00-fold with Aβ 1–42, ~1.35-fold with either Aβ 1–28 or Aβ 25–35, and no difference was found with Aβ 1–16 treatment (Figure 5D). These effects were more pronounced upon the treatment of A549 cells transfected with control siRNA with chelerythrine (Figure 4D). Relative to control A549 cells in the presence of chelerythrine, the concentration of ACh in the media decreased by ~1.90-fold with Aβ 1–40, ~2.60-fold with Aβ 1–42, and ~1.60-fold with either Aβ 1–28 or Aβ 25–35, with no difference found with Aβ 1–16 treatment (Figure 5D). These results suggest that inhibiting PKC leads to decreased ACh levels and that the ability of Aβ to decrease those levels is increased upon blocking PKC with chelerythrine in A549 cells (Figure 5D), highlighting a possible role for PKC in the Aβ-dependent decrease of ACh in the media of A549 cells. 

More modest effects but similar trends were found in A549 cells transfected with p53 siRNA (Figure 5E). Compared to untreated A549 cells transfected with p53 siRNA, treatment with chelerythrine resulted in ~1.20-fold decrease in the levels of ACh (Figure 5E). Relative to A549 cells transfected with p53 siRNA in the absence of chelerythrine, the levels of ACh decreased by ~1.40-fold with Aβ 1–40, ~1.55-fold with Aβ 1–42, ~1.20-fold with either Aβ 1–28 or Aβ 25–35, and no difference was found with Aβ 1–16 treatment (Figure 5E). These effects were more pronounced upon transfection of A549 cells with p53 siRNA and treatment with chelerythrine (Figure 5E). Relative to control A549 cells transfected with p53 siRNA in the presence of chelerythrine, the concentration of ACh in the media decreased by ~1.75-fold with Aβ 1–40, ~2.35-fold with Aβ 1–42, ~1.50-fold with either Aβ 1–28 or Aβ 25–35, and no difference was found with Aβ 1–16 treatment (Figure 5E). These results were comparable to those obtained using H1299 cells transfected with p53 siRNA (Figure 5F). 

Collectively, these results clearly show that PKC and p53 are important regulators of the levels of ACh. However, the observation that the levels of ACh are still reduced upon cell treatment with Aβ and blocking the PKC activity with chelerythrine in both cell lines along with p53 knockdown in A549 cells suggests that other pathways are involved in the regulation of the concentration of ACh in addition to p53 and PKC. Other kinases might also be involved, for example, cyclin dependent kinase-5 (Cdk5), since Aβ 25–35 was found to induce p53 phosphorylation and functional stabilization via activation of Cdk5, known to phosphorylate p53, leading to neuronal apoptosis [87].

### 2.6. Treatment of Cells with ACh Decreased the Activity of p53 in A549 Cells, Decreased p38 MAPK Activity, and Increased PKC Activity in Both A549 and H1299 Cells, Effects That Were Diminished by Cell Co-incubation with ACh and Aβ

**Figure 6:**
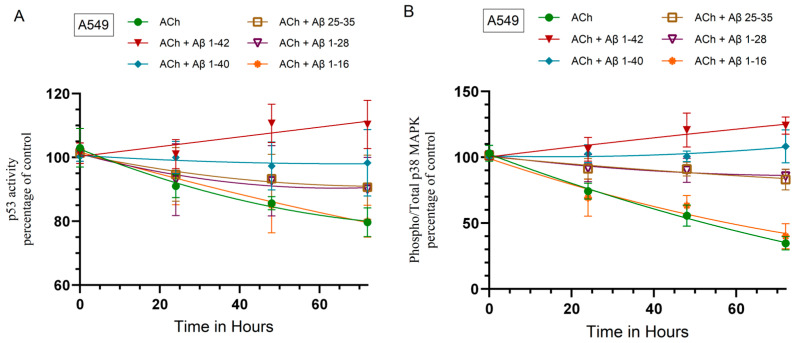

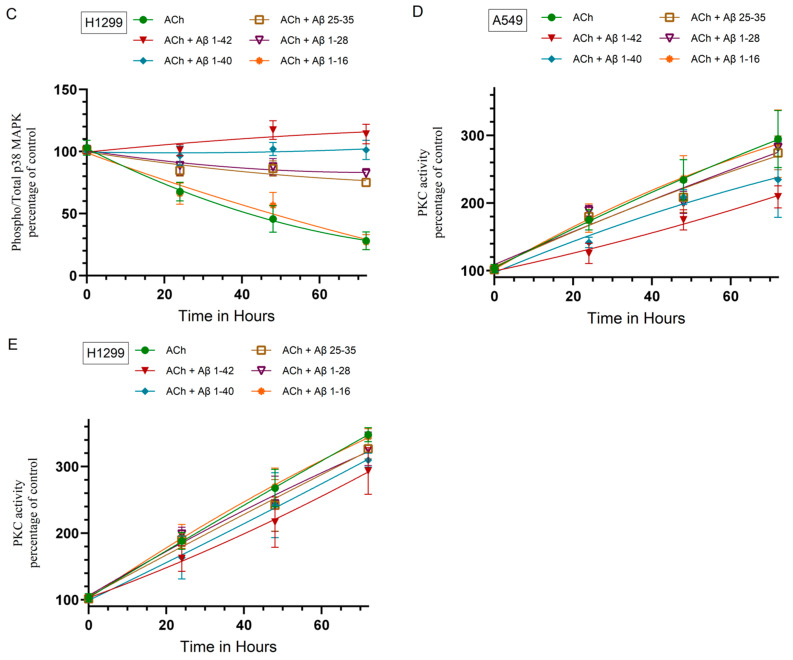
Incubation of cells with ACh reduced the activity of p53 in A549 cells, diminished the activity of p38 MAPK, and enhanced the PKC activity in both A549 and H1299 cells, while co-incubation with Aβ reduced those effects. Cells (0.2 × 10^5^) were grown in 10% FBS-supplemented media for 24 h and then serum-starved overnight (Control, 0 hour). The cells were then treated as indicated with ACh (100 nM) and in combination with Aβ 1–40/42 or fragments (10 μM). The p53 activity (**A**) in A549 cells and the activity of p38 MAPK (**B**,**C**) and PKC (**D**,**E**) were measured as described in the Materials and Methods Section. The data were expressed as the percentage of control by expressing each point relative to the control (set to 100%). The data were then plotted as a function of time using the GraphPad Prism 10.1.1 software. Data were expressed as the mean ± S.D. of three independent experiments, each carried out in triplicate.

We next tested the effect of exogenously added ACh on the activities of p53, p38 MAPK, and PKC in the absence or presence of Aβ 1–40/42 or fragments (Figure 6). Cells were grown in FBS-supplemented media overnight and then serum-starved for 24 h. The cells were then treated as indicated with ACh and in combination with Aβ 1–40/42 or fragments. The p53 activity in A549 cells and the activity of p38 MAPK and PKC were then measured as described in the Materials and Methods Section (Figure 6). 

The activity of p53 decreased ~20% after a 72 h incubation of A549 cells with either ACh alone or with both ACh and Aβ 1–16 (Figure 6A). Co-incubation of A549 cells with ACh and either Aβ 1–28 or Aβ 25–35 led to ~10% decrease in p53 activity and co-treatment with ACh and Aβ 1–42 led to ~10% increase in the activity of p53, while the effects of ACh were abolished by co-treatment with Aβ 1–40 and ACh (Figure 6A). These results show that, except for Aβ 1–16, Aβ 1–40/42 or fragments decreased the effects of ACh on the activity of p53 in A549 cells.

The phospho/total p38 MAPK ratio decreased by ~65% and ~73% after a 72 h incubation of A549 and H1299 cells with ACh alone, respectively (Figure 6B,C). Co-treatment of either A549 or H1299 cells with ACh + Aβ 1–16 had no effect compared to treatment with only ACh (Figure 6B,C). Co-treatment with ACh + either Aβ 1–28 or Aβ 25–35 decreased this ratio by ~14% in A549 cells (Figure 6B) and by ~20% in H1299 cells (Figure 6C). Co-treatment with ACh + Aβ 1–40 led to ~8% increase in the ratio of phospho/total p38 MAPK in A549 cells (Figure 6B) and abolished the effects of ACh in H1299 cells (Figure 6C). Co-treatment with ACh + Aβ 1–42 led to ~24% increase in the ratio of phospho/total p38 MAPK in A549 cells (Figure 6B) and ~14% increase in this ratio in H1299 cells (Figure 6C). These results show that ACh acts to decrease the ratio of phospho/total p38 MAPK and that addition of Aβ 1–40/42 or fragments, except for Aβ 1–16, counters the effects of ACh on the activity of p38 MAPK in A549 and H1299 cells.

Opposite effects were found on the PKC activity (Figure 6D,E) compared to those observed for the activities of p53 and p38 MAPK under the same conditions. There was a ~195% increase and a ~244% increase in the activity of PKC after a 72 h incubation of A549 and H1299 cells with ACh alone, respectively (Figure 6D,E). The effects of co-treatment of either A549 or H1299 cells with ACh + Aβ 1–16 on the activity of PKC were indistinguishable from those obtained by treatment with only ACh (Figure 6D,E). Co-treatment with ACh + either Aβ 1–28 or Aβ 25–35 increased PKC activity by ~174% in A549 cells (Figure 6D) and by ~227% in H1299 cells (Figure 6E). Co-treatment with ACh + Aβ 1–40 led to ~135% increase in PKC activity in A549 cells (Figure 6D) and ~210% increase in this activity in H1299 cells (Figure 6C). Co-treatment with ACh + Aβ 1–42 led to ~109% increase in the activity of PKC in A549 cells (Figure 6D) and ~193% increase in this activity in H1299 cells (Figure 6E). These results show that ACh acts to activate PKC and that addition of Aβ 1–40/42 or fragments, except for Aβ 1–16, counters the effects of ACh on the activation of PKC in A549 and H1299 cells. 

### 2.7. The Negative Effects of Aβ on Cell Viability Were Diminished by Cell Co-treatment with ACh

Aβ, well known for its association with AD, has been found to induce cytotoxic effects in a range of tumor cells in addition to brain cells [58,88,89]. A study showed that oligomeric Aβ 1–40 and Aβ 1–42 repressed breast cancer stem cell viability and pointed to ferroptosis as the main contributor to cell death induced by Aβ [90]. Cell viability assays also showed that Aβ 1–40 and Aβ 1–28 suppressed the viability of a panel of human squamous cell carcinoma cell lines [40]. 

**Figure 7:**
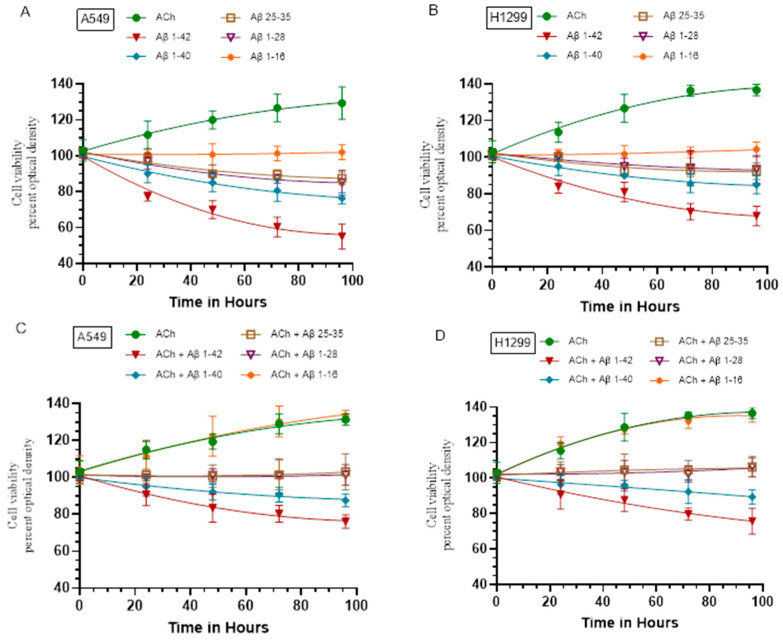
Treatment of cells with ACh diminished the negative effect of Aβ on cell viability. Cells (0.2 × 10^5^) were grown in 10% FBS-supplemented media for 24 h and then serum-starved overnight (control, 0 hour). The cell monolayers were then treated as indicated with ACh (100 nM), Aβ 1–40/42 or fragments (10 μM), and in combination, and then, cell viability (**A**–**D**) was measured and normalized to cell number (absorbance/cell number), as described in the Materials and Methods Section. Optical densities (570 nm) were normalized for the curves by expressing each point relative to the control (set to 100%). The data were then plotted as a function of time using the GraphPad Prism 10.1.1 software. Data were expressed as the mean ± S.D. of three independent experiments, each carried out in triplicate.

To examine whether Aβ 1–40/42 or fragments affect A549 and H1299 cell viability and the effects of added ACh, if any, cells were grown in 10% FBS-supplemented media for 24 h and then serum-starved overnight. The cell monolayers were then treated as indicated (Figure 7) with ACh, Aβ 1–40/42 or fragments, and in combination, and then, cell viability was measured as described in the Methods section. Cell viability increased by ~25% and ~35% after the incubation of A549 and H1299 cells with ACh, respectively (Figure 7A,B). These results are consistent with previous reports showing that ACh exerts its effects by binding to nicotinic and muscarinic receptors on lung cancer cells, thereby activating signaling cascades known to protect and accelerate cell survival and proliferation [38,39,40,41,47,51,91,92]. Incubation of either A549 or H1299 cells with Aβ 1–16 had no effect on cell viability while treatment with either Aβ 1–28 or Aβ 25–35 led to ~15% decrease in A549 cell viability and a ~5% decrease in H1299 cell viability (Figure 7A,B). Cell viability decreased by ~20% in A549 cells treated with Aβ 1–40 and by ~40% with Aβ 1–42 (Figure 7A). Treatment of H1299 cells with Aβ 1–40 decreased viability by ~14%, while ~30% decrease in viability was found upon treatment of H1299 cells with Aβ 1–42 (Figure 7B).

Co-treatment of A549 or H1299 cells with both ACh and Aβ 1–16 was indistinguishable from the observed effects on cell viability with cells treated with only ACh (Figure 7C,D). A549 and H1299 cell treatment with ACh and either Aβ 1–28 or Aβ 25–35 completely abolished the effects of the peptides on cell viability (Figure 7C, D) observed in the absence of ACh (Figure 7A,B). Cell viability decreased by ~10% in A549 cells treated with ACh + Aβ 1–40 and by ~20% in cells treated with ACh + Aβ 1–42 (Figure 7C), an effect diminished compared to cells not co-treated with ACh (Figure 7A). Similarly, co-incubation of H1299 cells with ACh + Aβ 1–40 decreased cell viability by ~8%, and ~20% decrease in cell viability was observed when H1299 cells were treated with both ACh and Aβ 1–42 (Figure 7D). These results show that ACh acts to diminish the effects of Aβ on A549 and H1299 cell viability. 

**Figure 8:**
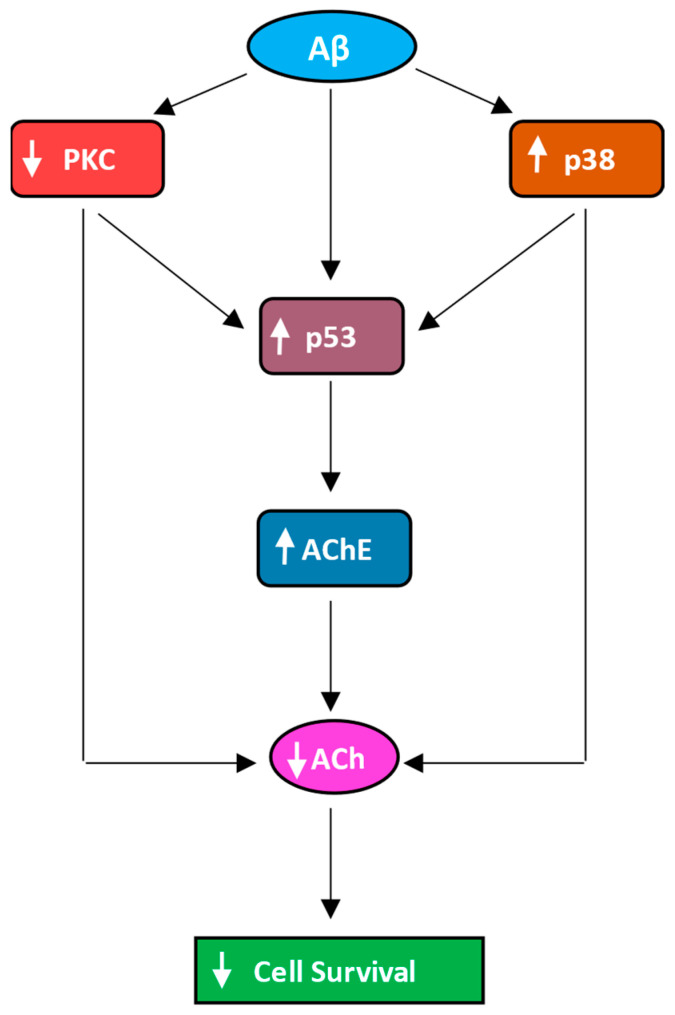
Representation of the main hypothesis and findings of this study. Aβ decreases the levels of ACh in the media via the activation of p53/AChE and p38 MAPK and/or via blocking the activity of PKC, leading to decreased cell survival. In this model, ACh reverses the effects of Aβ.

Based on our data, we propose a mechanism by which Aβ decreases the levels of ACh in the media of A549 and H1299 cells (Figure 8). 

Results from this study show that addition of Aβ leads to decreased levels of ACh in the media of NSCLC cells by the activation of p53/AChE and p38 MAPK and/or by blocking the activity of PKC, leading to diminished cell survival. This work also shows that ACh reverses the effects of Aβ. This study, however, does not address whether internalization of Aβ is an operative mechanism by which Aβ exerts these effects. Internalization of Aβ has been attributed to several pathways, and reuptake or entry of soluble Aβ 1–40/42 is thought to primarily occur by endocytosis [93]. Therefore, further work is needed to shed light on the extracellular and/or intracellular role of Aβ in this process in NSCLC cells.

AD and cancer are among the most devastating conditions that affect people today, and no effective treatment is currently available despite decades of intense research [5,11,13,94]. Some evidence suggests that people diagnosed with AD have a decreased risk for cancer and that those who survived cancer have a decreased risk for AD [11,13]. Therefore, innovative, paradigm-shifting views of the etiology of both cancer and AD are needed to enable their prevention and timely treatment. Aβ has been reported to be present in carcinoma cells and concentrated near blood vessels with significantly increased blood plasma peptide levels found in patients with melanoma, glioma, and adenocarcinoma, and in those with lung, breast, esophageal, colorectal, and hepatic cancers [5,16,95]. Findings from this study provide mechanisms that might shed light on new perspectives and understanding of how anti-cancer treatments might work in modifying the risk for AD and help in the development of therapeutic approaches for the prevention and treatment of cancer and AD in the future.

## 3. Materials and Methods

### 3.1. Materials

Most of the material used in this study was purchased as we reported earlier [4,53,68,96,97,98,99,100]. Aβ 1–40 (AS-24235), Aβ 1–42 (AS-20276), Aβ 1–28 (AS-24231), Aβ 25–35 (AS-24227), and Aβ 1–16 (AS-24225) were purchased from AnaSpec. Biotin-HN (HN, B-018--26, UniProt Q8IVG9) was purchased from Phoenix Pharmaceuticals. Phosphate Buffered Saline (PBS), nitrocellulose membranes, streptavidin–horseradish peroxidase (HRP) conjugate, Ponceau S solution, chelerythrine chloride, and ACh were purchased from Sigma-Aldrich. Mouse α-tubulin monoclonal antibody (DM1A), goat anti-rabbit IgG (H+L) secondary antibody (HRP, 31466), 3,3′,5,5′-tetramethylbenzidine (TMB), BCA protein assay kit, super signal west pico luminol (chemiluminescence) reagent, lipofectamine 2000 transfection reagent, and the Halt protease and phosphatase inhibitor cocktail were from ThermoFisher. Donkey anti-mouse IgG (HRP) (ab205724) was purchased from Abcam. m-IgGκ BP-HRP was obtained from Santa Cruz Biotechnology. SignalSilence p53 siRNA I (6231), SignalSilence control siRNA (Unconjugated, 6568), rabbit p53 antibody (9282), p38 MAPK antibody (9212) that detects endogenous levels of total p38α/β/γ MAPK, phospho-p38 MAPK (Thr180/Tyr182) antibody (9211), PKCα antibody (2056), and SB203580 (5633S) were purchased from Cell Signaling Technology (Danvers, MA, USA). 

### 3.2. Peptides Synthesis, Purification, and Characterization

Aβ 1–16, Aβ 1–28, and Aβ 25–35 were also synthesized on a 0.1 mmole scale via solid phase peptide synthesis using a PurePep Chorus automated peptide synthesizer from Gyros Protein Technologies (Lakewood, CO, USA). Rink amide MBHA resin was used as a solid support. The side chains of amino acids were protected as follows: Ser, Tyr, Asp, and Glu as the t-butyl derivatives (tBu), Arg with the 2,2,4,6,7-pentamethyIdlhydrobenzofuran-5-sulfonyl group (Pbf), Gln and Asn as trityl (Trt), and His and Lys as the t-butyloxycarbonyl (Boc) forms. N-a-Fluorenylmethoxycarbonyl (Fmoc)-protected amino acids were coupled in four-fold excess using *O*-(1*H*-6-Chlorobenzotriazole-1-yl)-1,1,3,3-tetramethyluronium hexafluorophosphate (HCTU) as an activating agent. Moreover, 20% piperidine in N,N-dimethylformamide (DMF) was used to deprotect the Fmoc group at each cycle. The peptides were side-chain-deprotected and cleaved from the resin using trifluoroacetic acid (TFA), distilled water, phenol, and triisopropylsilane (TIS), 88:5:5:2, for 2 h and precipitated with chilled diethyl ether, followed by vacuum filtration, aqueous dissolution, and lyophilization. Purification was carried out by reverse-phase HPLC using a Waters system with a 250 × 20 mm Higgins Analytical Proto300 C18 column, utilizing a gradient of water (0.1% TFA) to acetonitrile (0.1% TFA) and monitoring at 280 or 220 nm. Purity of combined lyophilized fractions was assessed by a Shimadzu analytical RP-HPLC with the same solvent system using a Phenomenex C18 column (Thermo Fisher Scientific, Waltham, MA, USA) (250 × 4.6 mm) at 220 nm. The molecular weight was determined using paper spray ionization mass spectrometry.

### 3.3. Cell Culture

The human NSCLC cell lines, A549 (ATCC CCL-185) and H1299 (ATCC CRL-5803), were purchased from the American Type Culture Collection (ATCC, Manassas, VA, USA). Cells were seeded as we reported earlier [34,53,56,68,69,96,97,98] in 5 mL DMEM/F12 media (GE Healthcare Life Sciences, Pittsburgh, PA, USA), supplemented with 10% FBS, 50 U/mL penicillin, and 50 U/mL streptomycin in 25 cm^2^ tissue culture flasks. The cells were allowed to grow overnight in an incubator at 37 °C, 95% humidity, and 5% CO_2_. The cells were counted using a hemocytometer after trypan blue staining. When inhibitors were used, cells were treated with inhibitors targeted against PKC (chelerythrine, 7.5 μM) or p38 MAPK (SB203580, 20 μM) as indicated.

### 3.4. MTT Assay

The MTT reduction assay (Sigma-Aldrich, St. Louis, MO, USA), used to measure cell viability, was carried out as we reported earlier [53,96,101,102]. The absorbance was measured at 570 nm in a plate reader. All absorbance measurements were in the linear range. Untreated cells or wells containing only DMSO and media were used as a positive and negative control, respectively. 

### 3.5. ELISA

ELISAs were conducted as we reported previously using Nunc MaxiSorp 96-well flat bottom plate (ThermoFisher) wells [2,4,99,100,102,103]. All absorbance measurements were in the linear range. To monitor non-specific binding, negative control wells on the plates included, for example, bound pure Aβ peptides and then, all components, streptavidin–horseradish peroxidase, and TMB were added, but not biotin-HN. Before analysis, the OD from the data was corrected for non-specific binding by subtracting the mean background absorbance of the negative controls. Statistical analysis was carried out using the GraphPad Prism 10.1.1 software. Data were expressed as the mean ± S.D. Three to five independent experiments were carried out in triplicate for each assay condition. 

### 3.6. Quantitation of ACh Concentrations

The concentration of ACh was measured using the choline/acetylcholine assay kit (ab65345) according to the manufacturer’s recommendation and as we reported earlier [4]. Briefly, media samples were added to wells followed by the addition of the choline reaction mix in the absence or presence of AChE. The absorbance was then measured at 570 nm after incubation for 30 min at RT using a microplate reader. The amount of ACh was calculated by subtracting choline from total choline (choline + ACh). 

### 3.7. AChE Activity

AChE activity in the conditioned media was assayed by the Ellman method using the AChE activity assay kit (MAK119) and according to our methods [4,53,56] and those previously reported [40,51]. This assay measures a colorimetric (412 nm) product formed from thiocholine, produced by AChE, which reacts with 5,5′-dithiobis (2-nitrobenzoic acid). One unit of AChE is the amount of enzyme that catalyzes the production of 1.0 µmole of thiocholine per min at pH 8 at 37 °C. The colorimetric product was proportional to the AChE activity present. 

### 3.8. p53 Transcription Factor Activity Assay 

The colorimetric BioVision’s p53 transcription factor activity assay (Catalog # K923-100) kit was used to measure the activity of p53 as we reported previously [56,99,100,102,103]. Briefly, cell lysates containing activated p53 were added to wells of a 96-well plate coated with double-stranded oligonucleotides. After allowing interaction with the oligonucleotides in the plate wells and washing, a p53 primary antibody was added followed by the addition of HRP-conjugated secondary antibodies. After addition of the TMB substrate, the color signal was developed and measured at 450 nm.

### 3.9. Western Blotting

Cell lysate samples were analyzed according to our previous protocols [4,98,103]. Briefly, attached live cells were harvested, and the cell pellet was resuspended in lysis buffer containing 1 mM PMSF and the Halt protease and phosphatase inhibitor cocktail (ThermoFisher). The samples were then briefly sonicated and centrifuged, and the supernatants were stored at −80 °C until further analysis. The protein concentration was measured using the BCA protein assay kit. Following methods that we reported earlier [4,98,99,103], samples were fractionated by SDS-PAGE on a 12% gel and then transferred to a nitrocellulose membrane. The membrane was then blocked, washed, incubated with the primary and secondary antibodies, developed using SuperSignal West Pico luminol (chemiluminescence) reagent, and imaged with a Bio-Rad molecular imager. 

### 3.10. p38 MAPK Assay

p38 is known to be activated by phosphorylation at Tyr-182 and Thr-180 [78]. The activation of p38 MAPK was monitored using the phospho-p38 α (T180/Y182) and total p38 ELISA kit (RayBiotech, Peachtree Corners, GA, USA). The assay uses a sandwich ELISA format to detect both total and phospho-p38 MAPK. Briefly, cell lysate samples were added to wells precoated with an immobilized antibody. After washing the wells, anti-p38 α MAPK antibodies were added to detect phosphorylated p38 (Thr180/Tyr182) or pan p38. After washing the unbound antibodies, HRP-conjugated IgG was added, and then, the color was developed following the addition of the TMB substrate solution. The color was in proportion to the amount of pan p38 or p38 (Thr180/Tyr182) bound. The intensity of the color was measured at 450 nm.

### 3.11. PKC Assay

The kinase activity assay kit (Abcam, Cambridge, UK, ab139437) was used to quantitate the PKC activity as we previously reported [4,56]. Briefly, a polyclonal antibody was used in this solid phase ELISA to detect the phosphorylation of a specific PKC synthetic peptide. 

### 3.12. SiRNA Transfection

Transfections were carried out according to our earlier methods [4,53,98,102,103]. Control siRNA or p53 siRNA were each mixed with lipofectamine 2000 transfection reagent diluted in Opti-MEM Media (ThermoFisher) according to instructions provided by the manufacturer, and then, the mixtures were added to the cells at a final concentration of 100 nM for each siRNA. Cells exposed to lipofectamine 2000 alone were used as a mock control. Transfection was carried out over a period of 96 hours; however, knockdown was optimally observed after 72 hours, a time point shown for all the results in this study. Each measurement represents the mean ± S.D. of three–five independent experiments, each performed in triplicate. 

### 3.13. Statistical Analysis

The analysis was carried out as we previously reported [34,53,68,97]. To evaluate the statistical differences, the Mann–Whitney and an ordinary one-way ANOVA followed by Tukey’s post hoc multiple comparison test were performed using the GraphPad Software, 10.1.1.

## Data Availability

The data presented in this study are available in this article.
